# Protective effects of vitamin D_3_ (cholecalciferol) on vancomycin-induced oxidative nephrotoxic damage in rats

**DOI:** 10.1080/13880209.2023.2204916

**Published:** 2023-05-04

**Authors:** Rouba Yasser Al-Sroji, Shaza Al-Laham, Ahmad Almandili

**Affiliations:** a Department of Pharmacology & Toxicology, Faculty of Pharmacy, Damascus University, Damascus, Syria; b Department of Histopathology, Faculty of Dentistry, Damascus University, Damascus, Syria

**Keywords:** COVID-19, nephrotoxicity, kidney function, urea, creatinine, oxidative stress, lipid peroxides, superoxide dismutase

## Abstract

**Context:**

Vancomycin (VCM), an important antibiotic against refractory infections, has been used to treat secondary infections in severe COVID-19 patients. Regrettably, VCM treatment has been associated with nephrotoxicity. Vitamin D_3_ can prevent nephrotoxicity through its antioxidant effect.

**Objective:**

This study tests the antioxidant effect of vitamin D_3_ in the prevention of VCM-induced nephrotoxicity.

**Materials and methods:**

Wistar Albino rats (21) were randomly divided into 3 groups: (A) control; (B) VCM 300 mg/kg daily for 1 week; and (C) VCM plus vitamin D_3_ 500 IU/kg daily for 2 weeks. All the rats were sacrificed and serum was separated to determine kidney function parameters. Their kidneys were also dissected for histological examination and for oxidative stress markers.

**Results:**

Lipid peroxidation, creatinine, and urea levels decreased significantly (*p* < 0.0001) in the vitamin D_3_-treated group (14.46, 84.11, 36.17%, respectively) compared to the VCM group that was given VCM (MIC<2 μg/mL) only. A significant increase was observed in superoxide dismutase levels in the vitamin D_3_-treated group (*p* < 0.05) compared to rats without treatment. Furthermore, kidney histopathology of the rats treated with vitamin D_3_ showed that dilatation, vacuolization and necrosis tubules decreased significantly (*p* < 0.05) compared with those in the VCM group. Glomerular injury, hyaline dystrophy, and inflammation improved significantly in the vitamin D_3_ group (*p* < 0.001, *p* < 0.05, *p* < 0.05, respectively) compared with the VCM group.

**Discussion and conclusions:**

Vitamin D_3_ can prevent VCM nephrotoxicity. Therefore, the appropriate dose of this vitamin must be determined, especially for those infected with COVID-19 and receiving VCM, to manage their secondary infections.

## Introduction

Vancomycin (VCM) is used to treat hospital-acquired methicillin-resistant *Staphylococcus aureus* infections (Jorgensen et al. 2020). Although the use of high-doses of VCM is not approved by regulatory authorities, this use is reaching 15–20 mg/L during the provision of health care due to emerging drug resistance (Martin et al. 2010). VCM is known to induce renal dysfunction, and nephrotoxicity related to VCM therapy has been reported at an overall rate of 16% and as high as 35% when combined with an aminoglycoside antibiotic. However, the nephrotoxic mechanism of VCM is uncertain (Cunha 1995). In various pathological conditions of toxic renal damage, reactive oxygen species (ROS) have been shown to contribute to cell damage (Nishino et al. 2002). The oxidative stress leads to a decrease in antioxidant enzymes, such as superoxide dismutase (SOD), and an activation of the inflammatory pathways, the most important of which is the nuclear factor-κB (NF-κB) pathway (de Jesus Soares et al. 2007). The main site of renal re-absorption of various substances, including VCM, is the proximal renal tubule. VCM-induced nephrotoxicity may occur in or around the proximal tubule cells (Appel et al. 1986; Beauchamp et al. 1990). The number of published articles examining VCM and induced nephrotoxicity has increased with time (Pais et al. 2020).

Acute kidney injury in COVID-19 patients could be related to various factors because the pathophysiology is not yet completely understood (Ronco et al. 2020). Severe COVID-19 is independently associated with an increased risk of acute kidney injury beyond premorbid conditions and age (See et al. 2021). Although avoiding VCM and NSAIDs is a potential way to prevent acute kidney injury in COVID-19 patients (See et al. 2021), VCM is necessary to avoid secondary infections in severe COVID-19 coronavirus patients (Yin et al. 2020). Secondary bacterial infections were observed in 31% of patients who required invasive mechanical ventilation (Yin et al. 2020). Common nosocomial pathogens are mainly Gram-positive bacteria, including methicillin-resistant *S. aureus* (MRSA), methicillin-resistant coagulase-negative *Staphylococci* (MRCNS) and *Enterococci species*, which mainly cause ventilator-associated pneumonia (Yin et al. 2020). VCM 15 mg/kg IV per 8–12 h was recommended for treating these infections (Yin et al. 2020). However, due to the VCM-associated nephrotoxicity and the narrow treatment window, sub-optimal VCM concentrations were prevalent, leading to insufficient antibacterial potency or increased risk of acute kidney injury (Yin et al. 2020). Therefore, it is necessary to find factors that reduce the nephrotoxicity associated with VCM, so we can use it more safely in patients.

Vitamin D_3_ is one of the fat-soluble vitamins (Cashman et al. 2014). Less than 30% of vitamin D_3_ can be obtained through diet (Holick 1996). Vitamin D_3_ found in foods can exist in two forms. The first is vitamin D_2_ (ergocalciferol), found in vegetable sources such as sun-dried mushrooms, and the second is vitamin D_3_ (cholecalciferol), found mostly in oil-rich fish. Both vitamin D_2_ and D_3_ go through hydroxylation twice to become the biologically active form, namely 1,25-dihydroxy vitamin D_3_ [1,25(OH)2D_3_ or calcitriol] (Deluca and Cantorna 2001).

The majority of vitamin D_3_ in the body is obtained through sunlight-initiated biosynthesis in the skin. When the skin is exposed to UVB radiation and thermal stimulation, a7-dehydrocholesterol is converted to pre-vitamin D_3_ and then to vitamin D_3_ (Zella and DeLuca 2003). First, vitamin D_3_ is converted to 25(OH)D in the liver by hydroxylation; then the second hydroxylation occurs in the kidneys, which produces 1,25(OH)_2_D_3_, which is the biologically active form of vitamin D_3_. It binds to the nuclear vitamin D receptor (VDR) or the plasma membrane VDR. The biological actions of 1,25 (OH)_2_D_3_ mediate control gene expression (Deluca and Cantorna 2001; Zella and DeLuca 2003). VitD_3_-VDR forms homodimers or heterodimers with the retinoid X receptor (RXR), then the homodimers or heterodimers bind to vitamin D_3_ response elements (VDRE). Thus, the expression of specific target genes is activated (Dulak et al. 2000). The VDR mediates both genomic and non-genomic actions of vitamin D_3_. These two kinds of actions are involved in physiological processes through regulating the transcriptional activity of target genes and activation of intracellular second messengers, respectively (Feghali and Wright 1997; Donato et al. 2009).

The most recognized role of vitamin D_3_ is maintaining phosphorus and calcium homeostasis (Cranney et al. 2007). However, studies in the past decades have revealed wide-ranging activities for vitamin D_3_ different from conventional activities. Some of these activities include the regulation of cardiovascular and renal functions and the modulation of immune responses (Nagpal et al. 2005; Bouillon et al. 2008). Over the last several years, studies have shown the importance of vitamin D_3_ in other areas, such as cell proliferation and differentiation as well as inflammatory processes (Munker et al. 1996; Cli 2011). It also has a potent anticancer effect, especially against digestive-system cancers (Giovannucci et al. 2006).

Vitamin D_3_ is an antioxidant agent, and the activation of the vitamin D_3_-VDR complex is associated with increased antioxidant activity. Several animal studies show a close relationship between vitamin D deficiency and increased oxidative stress (Zhong et al. 2014). The molecular mechanisms behind the actions of vitamin D_3_ in VCM-induced nephrotoxicity may be based on diminishing oxidative stress.

In pathophysiological conditions, overproduction of ROS, such as superoxide anion, hydrogen peroxide and hydroxyl radical, decreases anti-oxidative defenses and causes oxidative stress, which is implicated in the development of endothelial dysfunction. Vitamin D elicits antioxidant effects through the upregulating expression of anti-oxidative enzymes, including SOD, which can scavenge free radicals. In addition, the genetic action of vitamin D led to the expression of nuclear factor erythroid 2-related factor-2 (Nrf_2_), which is a key transcriptional factor that suppresses ROS production from its various sources and upregulates the expression of the antioxidants (Kim et al. 2020).

Vitamin D_3_ protects the renal tubule cells by targeting the NF-κB pathways (Tan et al. 2006), decreasing the production of pro-inflammatory factors and oxidative stress (Sun et al. 2014). Vitamin D_3_ induces a complex formation between VDR and p65 NF-κB. This interaction between VDR and p65 prevents NF-κB from binding to the DNA elements in the promoter of Regulated upon Activation Normal T cell Expressed and Secreted (RANTES) gene (Tan et al. 2008).

Therefore, the present study was designed to detect the protective effect of oral administration of vitamin D_3_ (500 IU/kg) to prevent VCM-induced nephrotoxicity in male rats. It has been observed that giving vitamin D_3_ before or during the treatment of COVID-19 reduces the severity of this disease (Annweiler et al. 2020). The prevention of adverse outcomes of COVID-19 by using vitamin D_3_ can be explained by its ability to repress or activate several genes which bind to the VDRE (Annweiler et al. 2020).

## Materials and methods

### Animals

Twenty-one male Wistar Albino rats (age 8–10 weeks, weight 200–250 g) were purchased from the Scientific Research Center, Damascus, Syria. They were acclimatized for one week before starting the study protocol. Rats were kept at controlled environmental conditions (temperature 23 ± 2 °C, humidity 55 ± 15%, under a 12 h light/dark cycle). They had free access to a standard commercial rat chow (pellet form, in the sack, Benghazi Animal Feed Company, Benghazi, Libya) and distilled water. The research was approved by Faculty of Pharmacy, Damascus University (protocol no./241/), and was conducted in accordance with the guidelines of the National Institutes of Health (NIH) for Care and Use of Laboratory Animals.

### Experimental design

The rats were randomly divided into three groups (*n* = 7): (A) control; (B) VCM; and (C) VCM plus vitamin D_3_. The overall treatment period with vitamin D_3_ was two weeks. Vitamin D_3_ was administered by itself at 24 h intervals in the first four days of the treatment period; then it was combined with VCM for 7 days (Emeka et al. 2021). Vitamin D_3_ treatment was repeated at 24 h intervals for 14 days (Elbassuoni et al. 2018).

### Sacrifice of animals

All rats were sacrificed on the 15th day under ethyl ether. Then, blood samples were collected from the vena cava. The whole blood samples were centrifuged at 1500 *g* at 4 °C for 10 min, and the plasma was separated and stored at −80 °C until processing for renal function tests. Kidneys were excised, immediately weighed and stored. One from each rat was stored in a 15% formalin solution for histopathological evaluation. The other was washed with a cold saline solution and stored at −80 °C until processing for biochemical analyses.

### Drugs

Vancomycin (Korea United Pharm, Korea) was injected intraperitoneally (IP) at a dose of 300 mg/kg daily for 1 week, which is the dosage reported to cause marked nephrotoxicity in rats (Ocak et al. 2007; Emeka et al. 2021).

Vitamin D_3_ (cholecalciferol, BASF, Germany) was administered orally at a dose of 500 IU/kg daily for 2 weeks (Elbassuoni et al. 2018). This product can produce a milky suspension in water.

Stock solutions were freshly prepared daily and used for feeding.

### Renal function tests

#### Serum creatinine concentration (Cr)

A commercially available kit was used to determine serum creatinine levels (creatinine assay kit, Biosystems, Barcelona, Spain). In the sample, creatinine reacts with picrate in an alkaline medium, forming a colored complex. The absorption of samples and standard were measured twice (after 30 and 90 sec) spectrophotometrically (Hitachi U-1800) at 500 nm. The concentrations were calculated accordingly. Results were expressed as mg/dL.

#### Serum urea concentration (BUN)

A commercially available kit was used to determine serum urea levels (Urea assay kit, Biosystems, Barcelona, Spain). The reactions described below show the production of urea in the samples, where a colored complex is produced that can be measured by spectrophotometry:

$$\mathrm{urea}+H_{2}O\;\vec{\mathrm{urease}}\;2NH_{4}^{+}+CO_{2}$$

$$NH_{4}^{+}+\mathrm{Salicylate}+\mathrm{NaClO}\;\vec{\mathrm{nitroprusside}}\;\mathrm{Indophenol}$$

600 nm was used to measure the absorption of both samples and the standard, and concentrations were calculated accordingly. Results were expressed as mg/dL.

### Kidney weight/body weight ratio (%)

After sacrificing the rats, each rat’s body and kidney weights were measured on the day of sacrifice, and then the kidney weight/body weight ratio was determined accurately for each case. Finally, the ratio was converted to a percentage.

### Oxidative stress markers

#### Tissue homogenizations

Kidney tissues (which were accurately weighed during sacrifice) were homogenized in a cold phosphate-buffered saline (pH 7.4, 50 mmol) to prepare a 10% tissue homogenate. The resultant suspension was divided into two parts. The first one was used for the determination of malondialdehyde (MDA), and the second part was centrifuged at 10,000 *g* for 20 min at 4 °C, and the supernatant was used for SOD activity measurement.

#### Lipid peroxidation (LPO)

Lipid peroxidation (assessed based on MDA production) in the tissue homogenates of the kidney was measured by determining the levels of thiobarbituric acid-reactive substances. A colorimetric reaction with thiobarbituric acid (TBA) is a highly sensitive indicator for evaluating the injury induced by ROS in kidney tissues are exposed to oxidative stress (Zemmouri et al. 2017). In brief, 0.5 mL of kidney tissue homogenate was mixed with 2 mL of TBA reagent containing (0.375% TBA, 15% trichloroacetic acid and 0.25 N HCl). Samples were boiled for 15 min, cooled and centrifuged. The absorbance of the supernatant was spectrophotometrically read at 532 nm, using an extinction coefficient of 1.56 × 10^5^/M cm. The final concentration of unknown sample/g tissue = 100 × μM LPO equivalent/g tissue (Ohkawa et al. 1979; Annouf et al. 2020).

#### Superoxide dismutase activity

The procedure is as follows. First, a certain amount of a pyrogallol solution (60 mmol in 1 mmol HCl, 37 °C) was mixed well with a Tris-HCl buffer (0.05 M, pH 7.4) containing 1 mM Na_2_EDTA. The volume was adjusted to 3000 μL using the buffer.

The A325 nm value of the mixture without a sample was measured every 30 s for 5 min at 37 °C. Second, we repeated the exact previous step with the addition of the sample. Enzyme activity, which matches the amount of enzyme that inhibits the auto-oxidation of pyrogallol by 50%, was calculated and expressed per mg of protein (Li 2012).

### Histopathological examination of kidneys

Kidneys obtained from all animals were decapsulated and sectioned longitudinally into two equally-sized pieces then fixed in a 15% buffered formalin solution for 24 h. The specimens were dehydrated in graded ethanol, cleared in xylene, and embedded in a paraffin wax. Serial sections that are 4 to 5 μm thick were cut using a microtome (Leica). Hematoxylin and eosin staining were used for histopathological examination using a light microscope with a camera connected to a computer for photographic documentation. A minimum of 10 fields for each kidney slide were assessed.

The results were scored semi-quantitatively and in descriptive form. The examinations focused on renal tubules for the presence of dilatation and vacuolization. Special attention was paid to the features indicating tissue necrosis. The severity of these lesions was determined using scores on a scale of Grade 0 (normal), Grade 1 (< 25% injury in tubular epithelium) (mild), Grade 2 (25–50% injury in tubular epithelium) (moderate), Grade 3 (50–75% injury in tubular epithelium) (severe), Grade 4 (complete necrosis) (very severe) (Yucel et al. 2019). This study also examined renal glomerular injury and hyaline dystrophies. The severity of these lesions was determined using scores on a scale of Grade 0: No injury, Grade 1: partial injury, and Grade 2: complete injury. Finally, the presence of inflammation and medullary vascular congestion was given Grade 1, and their absence was Grade 0.

### Statistical analysis

Statistical analysis was performed using GraphPad Prism software version 8.2 (San Diego, CA, USA). Numerical data were expressed as (mean ± standard error of the mean SEM). Data were evaluated by one-way analysis of variance (ANOVA), followed by Tukey’s test multiple comparisons. Histological analysis which used categorical ordinal data was evaluated by the nonparametric Mann–Whitney U test. The frequency of categorical binary data was evaluated using Fisher’s exact test. *P*-values <0.05 were considered as statistically significant.

## Results

### Macroscopic Evaluation

**Figure 1.**
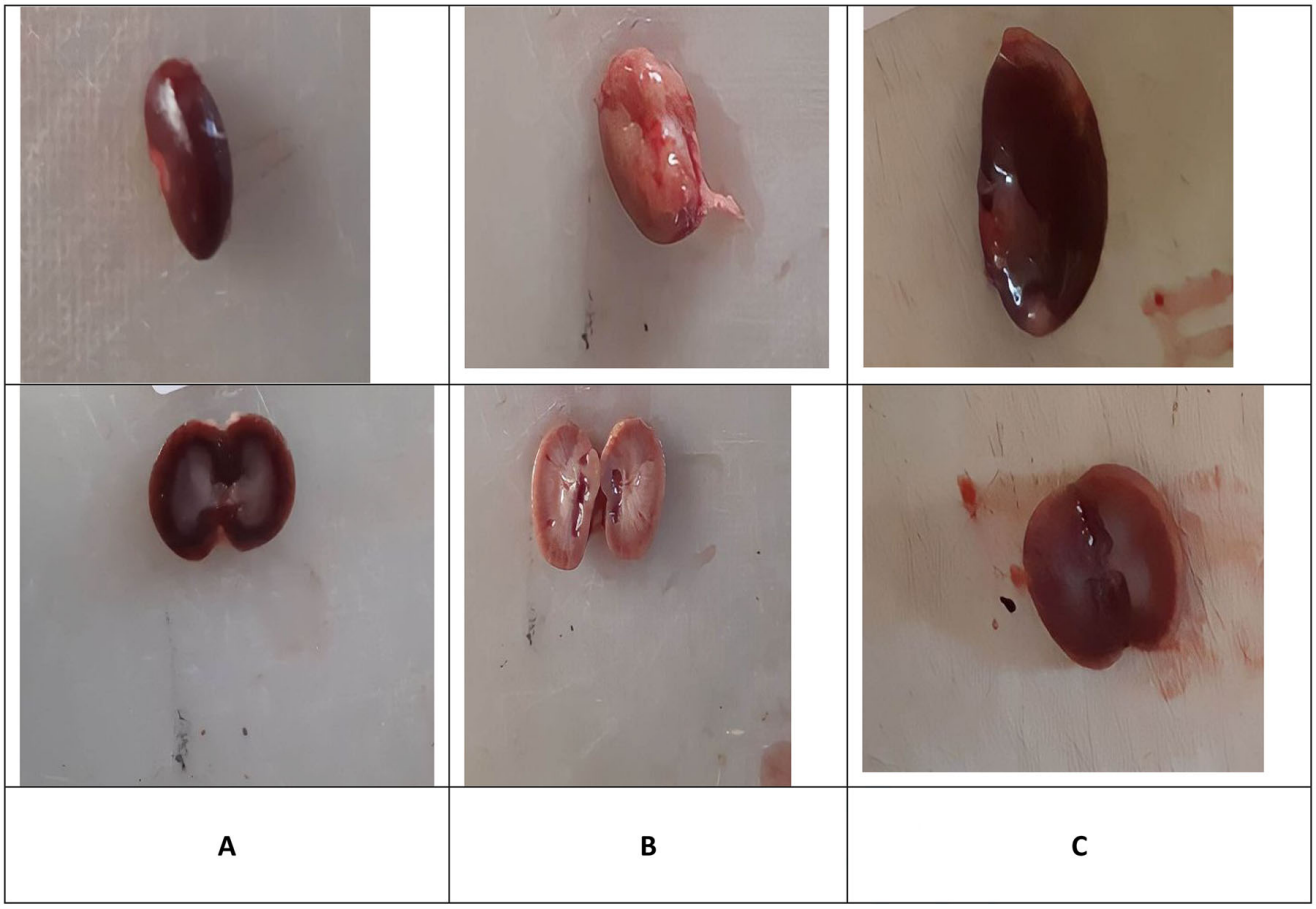
Macroscopically appearance of the kidney. Normal diet (A); vancomycin exposed group without treatment (B); vancomycin exposed group treated with vitamin D_3_ (C).

In the control group, kidneys had a normal macroscopic appearance. They were bean-shaped, surrounded by an easy-to-remove capsule. Their surface was smooth and red-brown in color. The sections showed the cortex and medulla, which were different in shade (Figure 1(A)). The kidneys in the VCM group were larger than those in the control group, and became pink and swollen with an unusual macroscopic morphology (Figure 1(B)). These morphological changes were markedly reversed in the vitamin D_3_ group, in which kidneys looked, to a certain extent, similar to those of the control group (Figure 1(C)).

### Kidney weight/body weight ratio (%)

**Figure 2.**
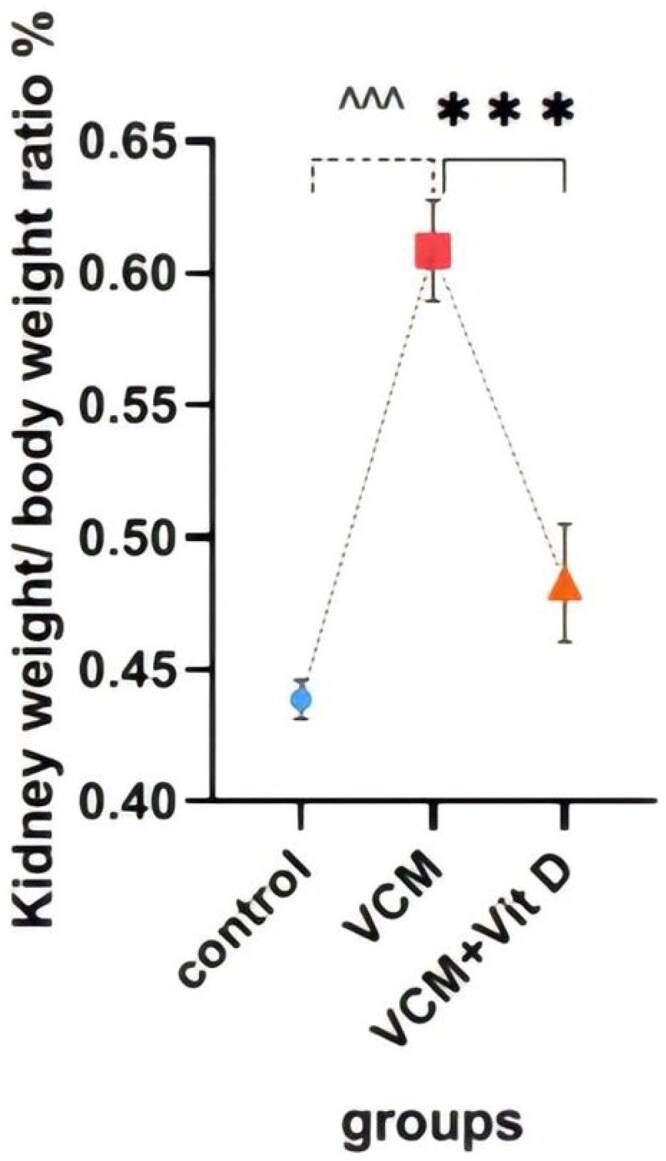
The effect of vancomycin and vitamin D3 on kidney weight/body weight ratio. The data are expressed in mean ± SEM and *n* = 7 in each group. Normal diet (control); vancomycin exposed group without treatment (VCM); vancomycin exposed group treated with vitamin D_3_ (VCM + Vit D) groups. ^^^*p* < 0.0001 compared with the corresponding value in the control group. ***p < 0.0001 compared with the corresponding value in the VCM group.

Table 1 shows the effects of VCM and vitamin D_3_ on the kidney weight/body weight ratio (%), as well as kidney function and oxidative stress markers. Administration of VCM alone (group B) significantly increased this ratio (*p* < 0.0001) compared with the control group (group A). However, this percentage was significantly lower (*p* < 0.0001) in the vitamin D_3_-treated group than in the VCM group (Figure 2).

**Table 1.** Vancomycin and vitamin D_3_ induced changes in kidney weight / body weight ratio (%), blood urea nitrogen (BUN), creatinine (Cr) levels in plasma, malondialdehyde (MDA) and superoxide dismutase (SOD) activity in kidney tissue in different rat groups.

	Groups		
Parameters	A	B	C
BUN (mg/dl)	30.36 ± 2.53	76.37 ± 1.89^a^	48.75 ± 1.70^b^
Cr (mg/dl)	0.14 ± 0.01	1.483 ± 0.06^a^	0.2357 ± 0.04^b^
MDA (µmol/100g)	2.70 ± 0.04	3.39 ± 0.07^a^	2.90 ± 0.07^b^
SOD activity	1.80 ± 0.03	1.11 ± 0.19^c^	1.53 ± 0.13^d^
Kidney weight/ body weight ratio (%)	0.43 ± 0.007	0.60 ± 0.01^a^	0.48 ± 0.02^b^

The data are expressed in mean ± SEM and *n* = 7 in each group. Normal diet (A); vancomycin exposed group without treatment (B); vancomycin exposed group treated with vitamin D_3_ (C).

*P*^a^ < 0.0001 versus control group.

*P*^b^ < 0.0001 versus vancomycin group.

*P*^c^ < 0.001 versus control group.

*P*^d^ < 0.05 versus vancomycin group.

### Biochemical results

**Figure 3.**
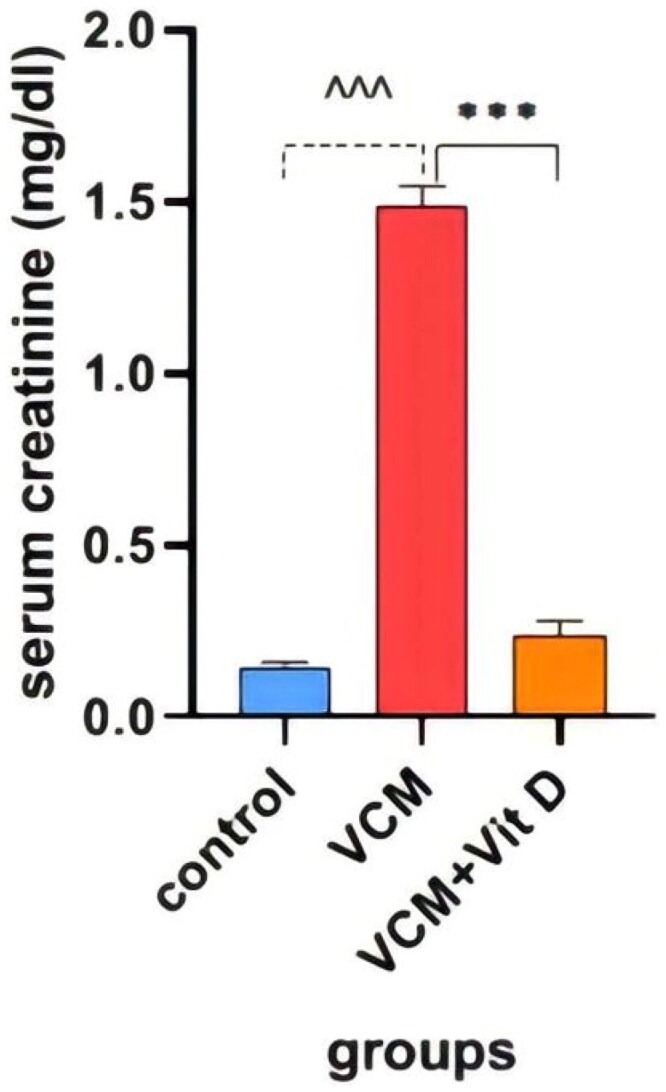
Effect of vancomycin and vitamin D_3_ on kidney function as creatinine levels in plasma. The data are expressed in mean ± SEM and *n* = 7 in each group. Normal diet (control); vancomycin exposed group without treatment (VCM); vancomycin exposed group treated with vitamin D_3_ (VCM + Vit D) groups. ^^^*p* < 0.0001 compared with the corresponding value in the control group. ***p < 0.0001 compared with the corresponding value in the VCM group.

**Figure 4.**
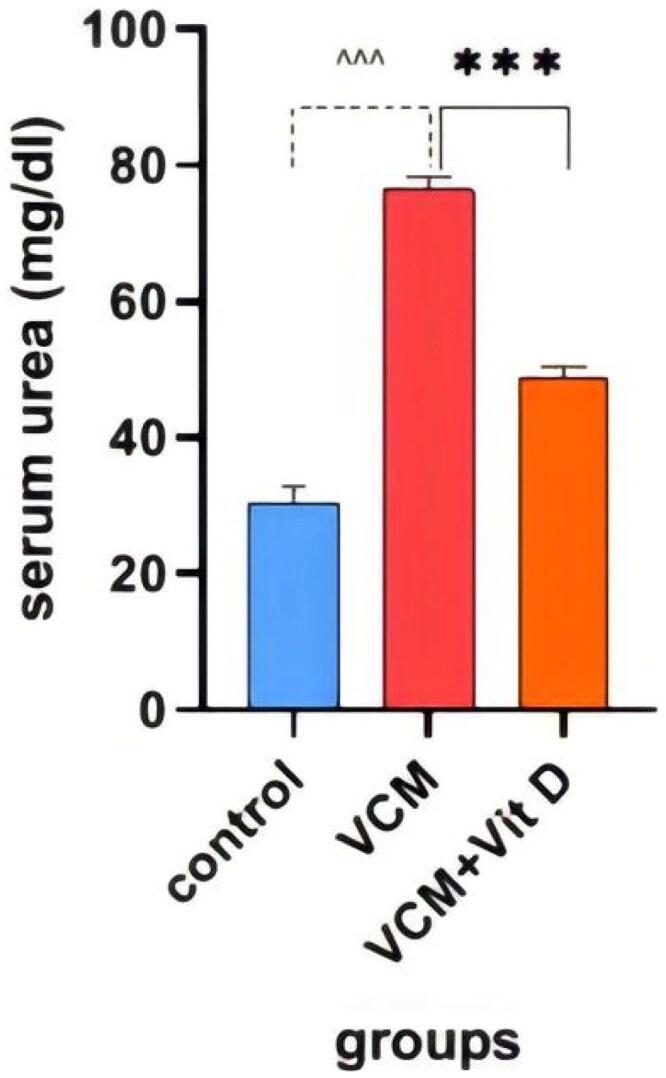
Effect of vancomycin and vitamin D_3_ on kidney function as urea levels in plasma. The data are expressed in mean ± SEM and *n* = 7 in each group. Normal diet (control); vancomycin exposed group without treatment (VCM); vancomycin exposed group treated with vitamin D_3_ (VCM + Vit D) groups. ^^^*p* < 0.0001 compared with the corresponding value in the control group. ***p < 0.0001 compared with the corresponding value in the VCM group.

**Figure 5.**
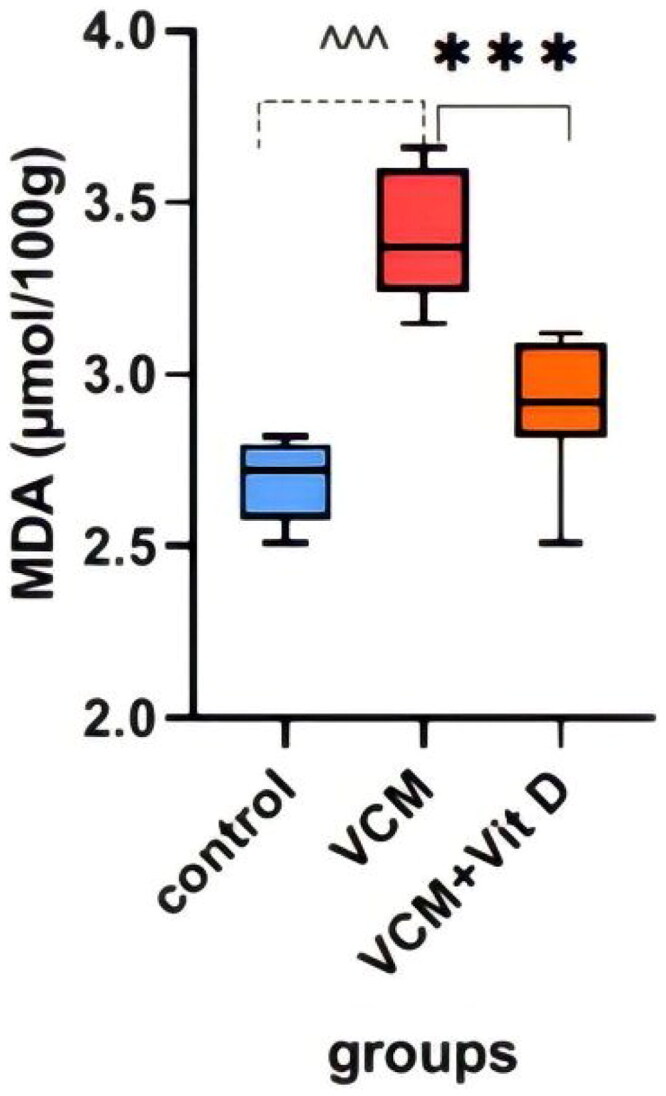
Effect of vancomycin and vitamin D_3_ on oxidative stress markers as malondialdehyde (MDA) levels in kidney homogenates. The data are expressed in mean ± SEM and *n* = 7 in each group. Normal diet (control); vancomycin exposed group without treatment (VCM); vancomycin exposed group treated with vitamin D_3_ (VCM + Vit D) groups. ^^^*p* < 0.0001 compared with the corresponding value in the control group. ***p < 0.0001 compared with the corresponding value in the VCM group.

**Figure 6.**
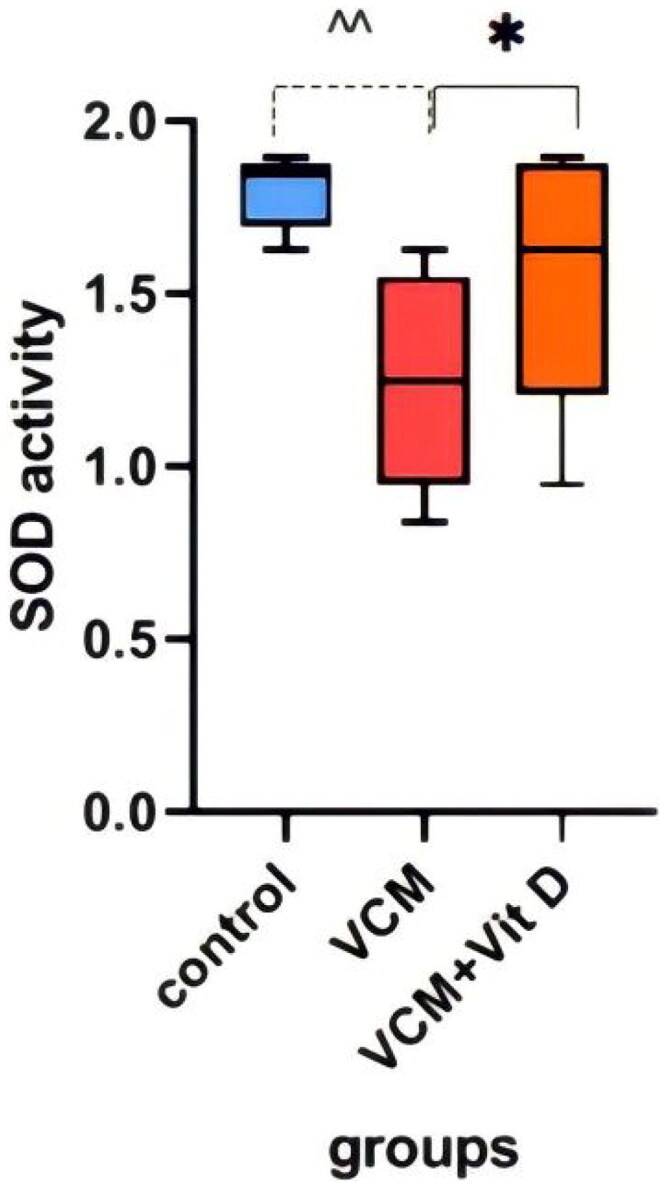
Effect of vancomycin and vitamin D_3_ on oxidative stress markers as superoxide dismutase activity (SOD activity) in kidney homogenates. The data are expressed in mean ± SEM and *n* = 7 in each group. Normal diet (control); vancomycin exposed group without treatment (VCM); vancomycin exposed group treated with vitamin D_3_ (VCM + Vit D) groups. ^^*p* < 0.001 compared with the corresponding value in the control group. *p < 0.05 compared with the corresponding value in the VCM group.

This study shows the effect of VCM and vitamin D_3_ on kidney function (urea, creatinine) in the serum of rats, as well as some oxidative stress biomarkers in kidney tissue. Administration of VCM (IP) resulted in a significant increase (*p* < 0.0001) in serum creatinine and urea of the group B (VCM-exposed group without treatment) compared to the control group (Table 1). In group C (VCM-exposed group treated with vitamin D_3_), the serum of creatinine significantly decreased (*p* < 0.0001) compared to the VCM group (Figure 3). Also, in the group of rats treated with vitamin D_3,_ the serum of urea significantly decreased (36.17%) (*p* < 0.0001) compared to group B (Figure 4). VCM-induced an oxidative cascade in rats’ Kidneys, the oxidative cascade was evaluated by LPO levels and SOD activity. There was a significant elevation in MDA levels in rats exposed to VCM compared to those in the control group (*p* < 0.0001). Antioxidant defenses significantly decreased in the VCM group, with the SOD activity in kidney tissues being lower than in the control group (*p* < 0.001). Supplementation with vitamin D_3_ (group C) resulted in a significant reduction (14.45%) of the MDA levels in kidney tissue (*p* < 0.0001) and a suitable increase (*p* < 0.05) in SOD activity compared to the VCM group (Figures 5 and 6).

### Microscopic Evaluation

**Figure 7.**
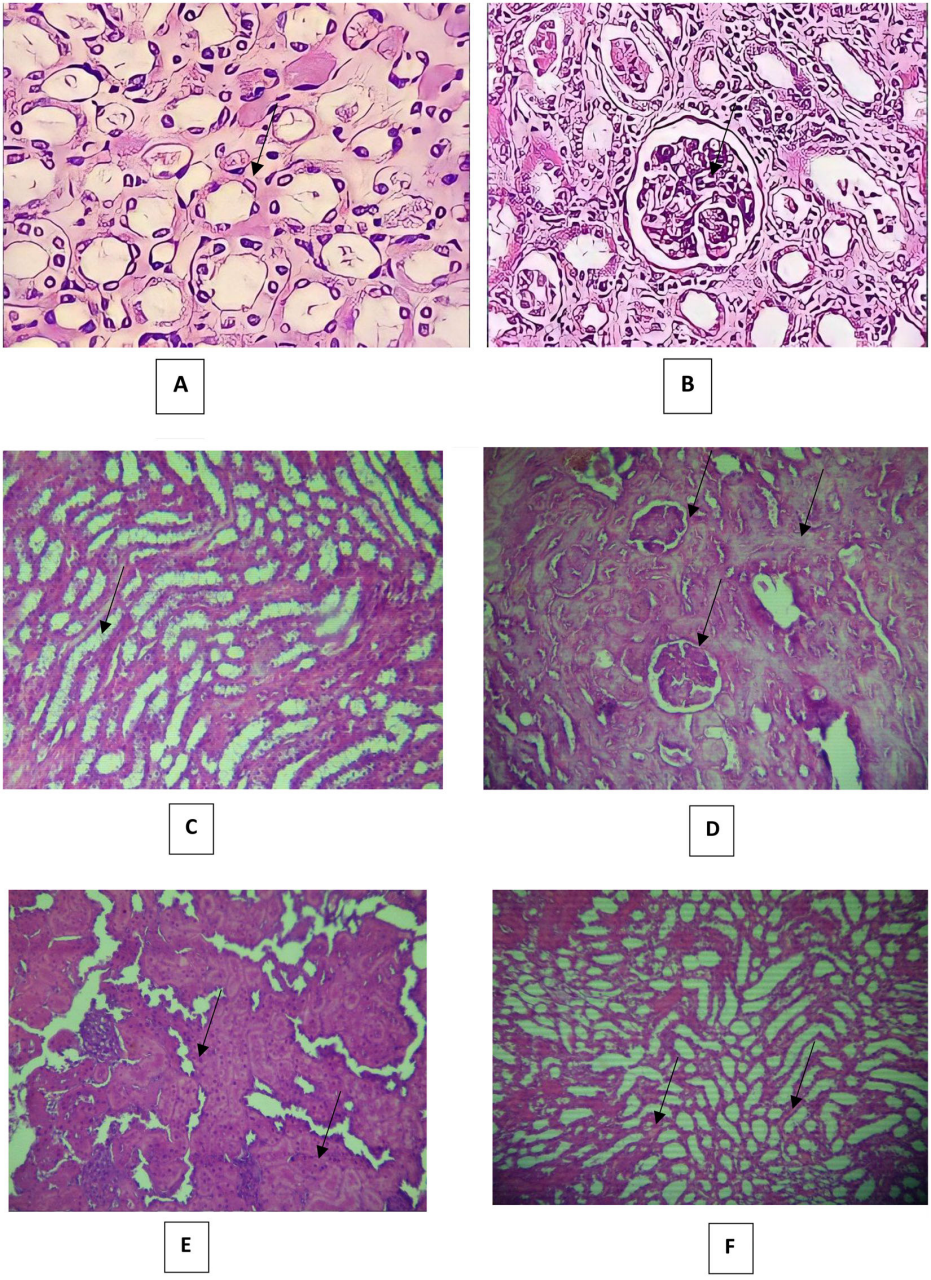
Histopathological changes in (300 mg/kg) dose vancomycin-induced renal injury in rats (Hematoxylin and eosin ×40). (A and B: control group). (A): normal renal tubules, (B): normal glomerulus; (C, D, E and F: vancomycin group). (C) abnormal dilatation of the renal tubules and vacuolization and necrosis of the epithelium of renal tubules, (D) complete glomerular injury and complete hyaline dystrophy, (E) Showing renal tubules filled with leukocytes, and (F) medullary vascular congestion.

**Figure 8.**
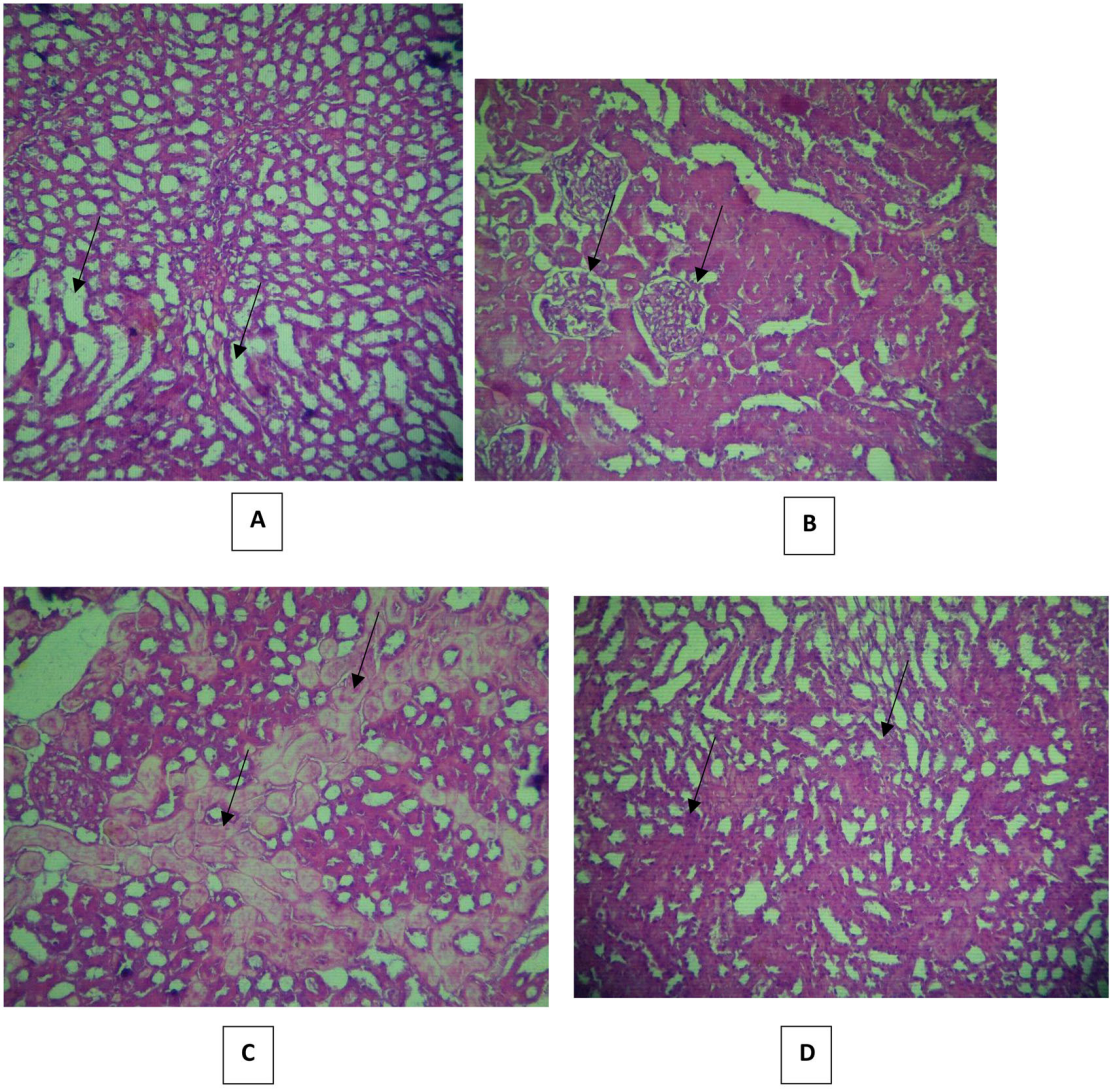
Effect of vitamin D_3_ (500 IU/Kg) on histopathological changes in histopathological changes in (300 mg/kg) dose vancomycin-induced renal injury in rats. (A, B, C and D: group C: hematoxylin and eosin ×40). In (A) Mild tubular dilatation and vacuolization tubules, (B) partial injury to the renal glomerular, (C) partial hyaline dystrophy and (D) Less distribution of leukocytes.

Histopathological changes in all groups’ kidneys were examined and scored, and the results are provided in Tables 2–4. Histology of the kidney sections of control animals showed normal. The renal tubules appeared normal: they were regular and with clearly-visible empty lumen without pathological deposits and were lined with one layer of cubic epithelium cells (Figure 7(A)). The glomerular and Bowman capsule appearances were normal (Figure 7(B)). Some specimens showed mild changes in the renal tubular histology and mild congestion. By contrast, severe lesions were seen in the VCM group’s kidney tubules, which showed dilatation, vacuolization, and typical necrosis morphology, including swelling, fragmentation and deformation of tubular epithelial cells (Figure 7(C)). Renal tubular scores were significantly higher (*p* < 0.001, *p* < 0.01, *p* < 0.01, respectively) compared with those of the control group (Table 2). Histopathological findings in rats without treatment (group B) showed mild to severe medullary congestion (Figure 7(F)). These injuries were significantly different (*p* < 0.001) from the group A (Table 4). The signs of glomerular injury were observed in the VCM group represented as mesangial extracellular matrix deformation, focal necrosis and glomerular capillary congestion (*p* < 0.001) compared with the control group (Figure 7(D) and Table 3). Hyaline dystrophies were also observed (Figure 7(D) and Table 3), unlike in the control group. Mononuclear and polymorphonuclear leukocyte infiltration was seen in the tubules and interstitium at high magnification (Figure 7(E) and Table 4). On the other hand, in rats treated with vitamin D_3_ (group C), the tubular lesions (tubular dilatation, vacuolization and necrosis) were significantly reduced (*p* < 0.05, *p* < 0.05, and *p* < 0.05, respectively) (Figure 8(A) and Table 2), and there was a significant amelioration (*p* < 0.001) in glomerular lesions compared with the VCM group (Figure 8(B), Table 3). Also, in rats treated with vitamin D_3_ (group C), a remarkable reduction of the histological inflammatory exudate was observed (*p* < 0.05) compared with rats without treatment (group B) (Figure 8(D), Table 4). The hyaline dystrophies were almost alleviated (*p* < 0.05) in the vitamin D_3_ group compared with the VCM group (Figure 8(C) and Table 3).

**Table 2.** Effects of vancomycin and vitamin D_3_ on histopathological scores of renal tubules (dilatation, vacuolization and necrosis) expressed as the frequency of injured rats in each group.

Groups	Grade 0	Grade 1	Grade 2	Grade 3	Grade 4	significance
	Histopathological Scores of dilatation tubules					
A	4 (57.15%)	2 (28.57%)	1 (14.28%)	0	0	
B	0	0	0	6 (85.72%)	1 (14.28%)	●●●
C	0	0	5 (71.43%)	2 (28.57%)	0	#
	Histopathological Scores of vacuolization tubules					
A	3 (42.86%)	2 (28.57%)	2 (28.57%)	0	0	
B	0	0	3 (42.86%)	2 (28.57%)	2 (28.57%)	●●
C	2 (28.57%)	0	5 (71.43%)	0	0	#
	Histopathological Scores of necrosis tubules					
A	3 (42.86%)	2 (28.57%)	2 (28.57%)	0	0	
B	0	0	2 (28.57%)	4 (57.14%)	1 (14.29%)	●●
C	0	2 (28.57%)	5 (71.43%)	0	0	#

Normal diet (A); vancomycin exposed group without treatment (B); vancomycin exposed group treated with vitamin D_3_ (C).

Grade 0 (normal).

Grade 1 (< 25% injury in tubular epithelium) (mild).

Grade 2 (25–50% injury in tubular epithelium) (moderate).

Grade 3 (50–75% injury in tubular epithelium) (severe).

Grade 4 (complete necrosis) (very severe).

●●●: *P*-value < 0.001 versus control group.

●●: *P*-value < 0.01 versus control group.

#: *P*-value < 0.05 versus vancomycin group.

**Table 3.** Effects of vancomycin and vitamin D_3_ on histopathological scores of glomerular injury and hyaline dystrophy expressed as the frequency of injured rats in each group.

Groups	Grade 0	Grade 1	Grade 2	Significance
	Histopathological Scores of glomerular injury			
A	7 (100%)	0	0	
B	0	0	7 (100%)	●●●
C	5 (71.43%)	2 (28.57%)	0	###
	Histopathological Scores of hyaline dystrophy			
A	7 (100%)	0	0	
B	0	0	7 (100%)	●●●
C	0	5 (71.43%)	2 (28.57%)	#

Normal diet (A); vancomycin exposed group without treatment (B); vancomycin exposed group treated with vitamin D_3_ (C).

Grade 0: No injury.

Grade 1: partial injury.

Grade 2: complete injury.

●●●: *P*-value < 0.001 versus control group.

###: *P*-value < 0.001 versus vancomycin group.

#: *P*-value < 0.05 versus vancomycin group.

**Table 4.** Effects of vancomycin and Vitamin D_3_ on histopathological scores of inflammation and medullary vascular congestion expressed as the frequency of injured rats in each group.

Groups	Grade 0	Grade 1	Significance
	Histopathological Scores of inflammation		
A	7 (100%)	0	
B	0	7 (100%)	●●●
C	5 (71.43%)	2 (28.57%)	#
	Histopathological Scores of medullary vascular congestion		
A	7 (100%)	0	
B	0	7 (100%)	●●●
C	2 (28.57%)	5 (71.43%)	ns

Normal diet (A); vancomycin exposed group without treatment (B); vancomycin exposed group treated with vitamin D_3_ (C).

Grade 0: no injury.

Grade 1: presence of an injury.

●●●: *P*-value < 0.001 versus control group.

#: *P*-value < 0.05 versus vancomycin group.

**ns**: *P*-value > 0.05 versus vancomycin group.

## Discussion

This study investigated the protective effect of vitamin D_3_ on VCM-induced nephrotoxicity. Oxidative stress markers (levels of MDA as well as activities of SOD) in kidney tissue and kidney function parameters (BUN and Cr) in serum were studied, and histopathological changes in renal tissue were also evaluated. Our study is the first trial in which the protective effect of vitamin D_3_ on VCM-induced nephrotoxicity was investigated based on biochemical and histological data.

VCM treats resistant infections, especially by Gram-positive bacteria. The VCM-induced nephrotoxicity occurs in 5–25% of treated patients (Iwamoto et al. 2003). In other studies, nephrotoxicity was up to 35% of patients (Aronoff et al. 1981). The data are less clear when the drug is used in combination with an aminoglycoside. It is known that the kidney is highly sensitive to toxic damage (Inoue et al. 1999). In recent studies, a significant increase in the levels of serum BUN and Cr were observed after treatment with VCM (Celik et al. 2005; Ocak et al. 2007). Levels of serum BUN is significant for the detection of late renal tissue damage, while levels of serum Cr are important for the detection of early renal failure (Erdem et al. 2000). In our study, levels of serum BUN and Cr in the VCM group was found to be significantly higher than those of the control group (*p* < 0.0001 for both of them). These results pointed out that VCM administration causes serious nephrotoxicity in rat models.

Previous studies also showed an increase in urea and creatinine levels when VCM was administered (Cetin et al. 2007; Qu et al. 2019; Malkani et al. 2020). The administration of vitamin D_3_ reduced urea and creatinine levels and protected against lipopolysaccharide-induced acute kidney injury (Xu et al.^2015^). In our study, levels of BUN and Cr in the vitamin D_3_-treated group were found to be less than those of the VCM group (*p* < 0.0001 for both of them), and these results are consistent with the study of Elbassuoni et al. (2018) but contradict the results of another study (Hur et al. 2013) in which vitamin D had no obvious effect on gentamicin-induced acute kidney injury in rats and did not reduce the rat’s urea and creatinine levels. The reason could be the difference in the route of administration of vitamin D and the short duration of administration in addition to the mechanism of action of gentamycin in acute kidney injury events.

Studies have shown various mechanisms for VCM-induced renal injury, suggesting that it could be multifactorial, involving several signaling pathways (Qu et al. 2019). VCM-induced apoptosis in LLC-PK1 cells by increasing intracellular ROS generation and by causing mitochondrial membrane depolarization, followed by caspase-9 activation and 3/7 (Arimura et al. 2012).

VCM treatment leads to tissue damage by elevated expression of NF-κB p65 in a dose-dependent manner (Qu et al. 2019). As well as the production of the pro-inflammatory cytokines IL-1 and TNF-α (Qu et al. 2019). Inflammation was significantly higher in the VCM group (*p* < 0.001) compared to the control group. This is consistent with the studies of Cetin et al. (2007), Ocak et al. (2007), and Emeka et al. (2021).

The molecular mechanism of VCM-induced nephrotoxicity could be associated with the inhibition of the signaling pathway of Nuclear factor erythroid 2-related factor 2 (Nfr_2_) (Emeka et al. 2021).

Nephrotoxicity could be caused by the accumulation of VCM in renal cells. We studied the nephrotoxicity of a single dose of VCM to investigate oxidant-antioxidant systems and pathological changes in the renal parenchyma. When different doses of VCM 50–400 mg/kg/day were administered, a similar decrease in creatinine clearance was observed, but the highest VCM doses caused mild histological changes (Aronoff et al. 1981). VCM administration at doses of 200–400 mg/kg twice a day showed that the plasma levels of urea and creatinine were significantly increased. It was also found that the glomeruli were destroyed, and the proximal tubular cells became swollen with obvious necrosis (Nishino et al. 2002).

In this study, the urea and creatinine levels increased significantly above those of the control group when VCM was administered at 300 mg/kg/day. VCM stimulated free radical production and oxidative stress by increasing oxygen consumption and cellular ATP concentration (King and Smith 2004). Free radicals can cause cellular injury, DNA damage, peroxidation of membrane lipids and protein denaturation *via* various mechanisms (Dean et al. 1991). Lipid peroxidation leads to damage to the structure and function of the membrane. This damage results in the generation of various end products, such as MDA (Vardi et al. 2005). Thus, there may be a direct proportion between MDA level and lipid peroxidation. Accordingly, the increase in the MDA level is accepted as an indicator of an increase in lipid peroxidation (Nielsen et al. 1997).

Previous studies showed that VCM increases the level of MDA in experimental animals (Basarslan et al. 2012; Emeka et al. 2021). When levels of MDA in the VCM group were compared with those of the control group, a significant increase was observed in the VCM group (*p* < 0.0001), and this indicates that lipid peroxidation was involved in the pathogenesis of nephrotoxicity induced by VCM. These results support previous studies by Nishino et al. (2003), Öktem et al. (2005), and Cetin et al. (2007) which reported that the administration of VCM intraperitoneally at a dose range of 200–400 mg/kg for 7 days led to a significant increase in the levels of MDA in VCM-induced nephrotoxicity.

Previous studies have reported that vitamin D reduces oxidative stress in the liver of streptozotocin-induced diabetic rats. A significant decrease in the level of MDA was observed (George et al. 2012). In our study, we found that vitamin D_3_ was able to create a retrospective effect by decreasing lipid peroxidation as the level of MDA in the vitamin D_3_-treated group was found to be significantly less (*p* < 0.0001) than that of the VCM group and this result is consistent with Elbassuoni et al. (2018), Tohari et al. (2019), and Mokhtari-Zaer et al. (2020). Our results also confirm the results of (Seif and Abdelwahed 2014) in which vitamin D succeeded in reducing both hepatic ischemia/reperfusion injury and the MDA level in the vitamin D_3_-treated group by reducing oxidative stress.

Cells have protective enzymes and antioxidant molecules, such as SOD, glutathione peroxidase and catalase. Over release of free radicals may surpass the antioxidative capacity of biological systems and lead to serious cellular damage (Reiter et al. 1993). SOD is the most important protective enzyme against oxidative stress in renal tubules (Inoue et al. 1999).

A significant decrease in the activities of SOD was found in renal injury induced by VCM (Celik et al. 2005; Ahmida 2012; Qu et al. 2019). A similar result was observed in our study. A more significant decrease (*p* < 0.001) in the activities of SOD was found in the VCM group than in the control group.

Previous studies indicated that vitamin D is able to protect the retina and retinal Pigment Epithelial Cells from oxidative stress by enhancing the antioxidant defense capacity, including that of SOD, as the level of SOD was significantly increased when vitamin D was given (Tohari et al. 2019). Remarkably, our study showed that the activity of SOD was significantly higher in the vitamin D_3_-treated group (*p* < 0.05) compared to the VCM group. The same results have been reported by other investigators in experimental animals (Mokhtari-Zaer et al. 2020), in which SOD increased in the vitamin D_3_-treated group, which improved the cognitive impairments that occurred due to lipopolysaccharide.

Recent studies emphasized that antioxidant substances provided a renal protective effect by diminishing lipid peroxidation (King and Smith 2004) and suggested that vitamin D_3_ reduced the production of superoxide anion on endothelial cells through the regulation of mediators of antioxidant activity, such as nuclear factor erythroid 2-related factor-2 and nuclear transcription factor κB (Teixeira et al. 2017).

Vitamin D_3_ displays anti-inflammatory activity by inhibiting RANTES and TNF-α expression in a mouse model. It mediates pro-inflammatory responses by the NF-κB pathway specifically *via* the binding of vitamin D_3_ -VDR complex to the p65 subunit so it prevents NF-κB from interacting with DNA elements (Tan et al. 2008). It is also likely that vitamin D_3_-VDR attenuates the expression of the P53-Upregulated Modulator of Apoptosis (PUMA) and miR-155 in tubular epithelial cells by disrupting NF-κB activation (Du et al. 2019).

ROS Stimulates the oxidation of proteins, DNA and lipid *via* the activation of NF-κB, which in turn activates the inflammation cascade by activating inflammatory cytokines, such as TNF-α. Activated NF-κB pathways contribute to acute kidney injury. Active vitamin D_3_ could attenuate glomerular injury and renal injury (Makibayashi et al. 2001; Tan et al. 2007) and it reduces oxidative stress. These effects may be due to enhancement in the cytosolic SOD enzyme and inhibition of NADPH oxidase expression (Finch et al. 2012).

One possible mechanism used by vitamin D_3_ to protect against VCM-induced nephropathy is the Nrf_2_–Keap1 pathway (Nakai et al. 2014). Nrf_2_ controls the expression of ROS and the antioxidant agents *via* the antioxidant response element (ARE/EpRE). In physiological conditions, Nrf_2_ is sequestered in the cytoplasm by the repressor protein Keap1. Owing to this mechanism, Keap1 contributes to augmented oxidative stress due to the negative regulation of Nrf_2_ and ARE/EpRE activity (Kobayashi and Yamamoto 2005). Vitamin D_3_ could increase the expression of Nrf_2_ and also leads to reduce expression of Keap1. That decreases the development of nephropathy by inhibition of oxidative stress (Nakai et al. 2014).

Our results in the kidney were in agreement with other studies on different organs. Vitamin D_3_ is reported to contribute to the prevention of some chronic diseases, such as diabetes (Gren 2013) and cardiovascular disease (El-Gohary and Allam 2017) by regulation of oxidative stress *via* increasing the antioxidant enzymes, such as glutathione peroxidase (GPx) and SOD, and suppressing the expression of NADPH oxidase. The increase in the activities of SOD and the decrease in MDA levels were detected after administering vitamin D_3_ to renal injury induced by VCM. Furthermore, histopathological evaluations supported these findings. All results indicate that vitamin D_3_ has an antioxidant effect on renal damage which was induced by VCM. Reports showed that VCM-induced nephrotoxicity target the proximal tubules (Beauchamp et al. 1990).

In our study, VCM-related nephrotoxicity is associated with tubular dilatation, tubular vacuolization and necrosis. These results are consistent with the results of the study (Cetin et al. 2007; Basarslan et al. 2012; Qu et al. 2019). The glomeruli in the kidney of the VCM group appeared abnormal compared to the control group, and this is similar to the results of the study (Celik et al. 2005; Ocak et al. 2007; Malkani et al. 2020). These injuries were significantly improved by giving vitamin D_3_. These results demonstrated that VCM-induced nephrotoxicity could be ameliorated by vitamin D_3,_ which was a retrospective agent that lessens toxic damage of VCM. Vitamin D_3_ may show this effect by decreasing LPO and activating the antioxidant system in the renal tubular cells.

## Conclusions

This study proved that VCM-induced kidney damage by raising the levels of serum BUN, Cr and MDA and that vitamin D_3_ protected the kidney by diminishing the levels of these compounds and increasing the levels of SOD. Vitamin D_3_ had a marked positive effect on VCM-induced nephrotoxicity and it ameliorated the oxidative status, biochemical damage and histopathological changes. As a result, we think that vitamin D_3_ may have a useful role as a novel retrospective agent for preventing the nephrotoxic damage of VCM if tested on COVID-19 patients. In our study, vitamin D_3_ dose and experiment duration were limited. Therefore, we suggest that future clinical studies should focus on the optimal dose of vitamin D_3_ and the suitable duration in humans for this antioxidant effect. Additionally, studies should be carried out to determine the exact mechanism of action that vitamin D_3_ has which deters this nephrotoxicity induced by VCM.
